# N6-Methyladenosine Induced miR-34a-5p Promotes TNF-α-Induced Nucleus Pulposus Cell Senescence by Targeting SIRT1

**DOI:** 10.3389/fcell.2021.642437

**Published:** 2021-03-05

**Authors:** Hao Zhu, Bao Sun, Liang Zhu, Guoyou Zou, Qiang Shen

**Affiliations:** ^1^Department of Orthopaedics, The Affiliated Shanghai General Hospital of Nanjing Medical University, Shanghai, China; ^2^Department of Orthopaedics, Yancheng First Hospital, Affiliated Hospital of Nanjing University Medical School, Yancheng, China

**Keywords:** N6-methyladenosine, IVDD, miR-34a-5p, cell senescence, SIRT1

## Abstract

Low back pain is tightly associated with intervertebral disc degeneration (IVDD) and aberrant nucleus pulposus (NP) is a critical cause. miRNAs N6-methyladenosine (m6A) modification accounts for the TNF-α-induced senescence of NP cells. The aim of this study was to investigate whether m6A modification regulates TNF-α-mediated cell viability, cell cycle arrest, and cell senescence and how it works. The results showed that METTL14 expression positively correlated with m6A and TNF-α expression in HNPCs. The knockdown of METTL14 led to the inhibition of the TNF-α-induced cell senescence. METTL14 overexpression promoted cell senescence. METTL14 regulated the m6A modification of miR-34a-5p and interacted with DGCR8 to process miR-34a-5p. The miR-34a-5p inhibitor inhibited the cell cycle senescence of HNPCs. miR-34a-5p was predicted to interact with the SIRT1 mRNA. SIRT1 overexpression counteracted the miR-34a-5p-promoted cell senescence. METTL14 participates in the TNF-α-induced m6A modification of miR-34a-5p to promote cell senescence in HNPCs and NP cells of IVDD patients. Downregulation of either METTL14 expression or miR-34a-5p leads to the inhibition of cell cycle arrest and senescence. SIRT1 mRNA is an effective binding target of miR-34a-5p, and SIRT1 overexpression mitigates the cell cycle arrest and senescence caused by miR-34a-5p.

## Introduction

Lower back pain (LBP) is the most common chronic pain that affects at least 80% of Americans in their lifetime (Freburger et al., 2009). In general, LBP can be caused by a muscle sprain or strain injury as well as certain diseases, including spinal cord cancer, ruptured or herniated disc sciatica arthritis, kidney infections, and spine infections. It has been revealed that LBP is strongly associated with intervertebral disc degeneration (IVDD) and degenerative disc disease is identified to be the main cause of LBP (Luoma et al., 2000; Morgan et al., 2014). However, despite some reports of treatment to mitigate the pain and symptoms, no effective therapeutic regimen toward IVDD has been established considering that LBP is multifactorial (Froud et al., 2014).

Intervertebral disc mainly comprises of inner nucleus pulposus (NP) and surrounded annulus fibrosus, in which NP is the inner core of the vertebral disc (Pattappa et al., 2012). Comprised of a jelly-like material mainly formed by water and a loose collagen fiber network, NP is essential to maintain intervertebral disc height and mechanical properties (Frost et al., 2019). NP is also characterized to be responsible for controlling the synthesis and decomposition of the NP extracellular matrix (ECM) (Hwang et al., 2014). Moreover, abnormal apoptosis of NP cells (NPCs) is correlated with the pathology of IVDD. The degeneration was mainly manifested by the apoptosis of NP cells, in which the levels of cleaved caspase-3 and Bax were upregulated while the expression of Bcl-2 was downregulated (Kepler et al., 2013; Jiao et al., 2018; Li Z. et al., 2019). Aberrant apoptosis and senescence of NP cells play significant roles in the process of IVDD (Zhao et al., 2006; Jiang et al., 2013; Chen et al., 2018). During the apoptosis of NPCs, tumor necrosis factor (TNF)-α represents a key pro-inflammatory cytokine to promote the induction of apoptosis (Xie et al., 2019). Thus, TNF-α has been commonly recognized as a contributor to IVDD (Wang et al., 2017). Additionally, researchers have unveiled the role of TNF-α in the premature senescence of rat NP cells (Li P. et al., 2017). Through the PI3K/Akt signaling, TNF-α promoted senescence of NP cells.

Non-coding RNAs (ncRNAs), including microRNAs (miRNAs), long non-coding RNAs (lncRNAs), and circular RNAs, have critical contributions to IVDD (Li et al., 2018; Zhu et al., 2019; Xiang et al., 2020). They participate in the regulation of the proliferation of human NP cells (HNPCs) and the synthesis of ECM as well as the degradation-regeneration balance of the ECM in IVDD. Recently, a comprehensive analysis of altered methylation level of miRNA and lnRNAs in IVDD patients was conducted, which verified that N6-methyladenosine (m6A) methylation was one of the most abundant internal RNA modifications in IVDD (Wang X. et al., 2020). A growing number of studies have characterized the critical role of mRNA m6A modification in human diseases (Weng et al., 2018; Han et al., 2019; Han et al., 2020). However, the role of this modification in the pathogenesis of IVDD is fully mysterious. Additionally, the silent mating type information regulator 2 homolog-1 (SIRT1) played a protective role in IVDD and preserves the normal NP cell phenotype (Zhang et al., 2011; Feng et al., 2016; Wang Y. et al., 2020). The mechanism behind has not been explicated yet. In this study, we report the Methyltransferase like (METTL)14-dependent m6A methylation of miR-34a-5p in IVDD patients. Through regulating the processing of miR-34a-5p that targeted SIRT1, METTL14-dependent m6A methylation promoted the TNF-α-induced cell senescence of human NP cells (HNPCs). Our research provided novel insights into the mechanism of IVDD development.

## Materials and Methods

### Clinical Sample Collection

The study was approved by the medical ethics committee of The Affiliated Shanghai General Hospital of Nanjing Medical University and was conducted in accordance with the Declaration of Helsinki. Written informed consents were obtained from all participants. From March 2015 to April 2018, degenerative nucleus pulposus (NP) samples were collected from 30 patients with intervertebral disc degeneration (IVDD) of low (0–3), moderate (4–6), and high (8–10) degenerative grades (*n* = 10 per group) using the Rutgers score (Nakazawa et al., 2018), who underwent operations at The Affiliated Shanghai General Hospital of Nanjing Medical University. Normal NP samples were collected as controls from ten volunteers. The NP tissues were prepared using the culture medium followed by washing with phosphate-buffered saline under sterile conditions. The prepared samples were then used for the qRT-PCR assay.

### Cell Culture and Treatment

HNPCs were isolated and cultured as per the previous report (Dudek et al., 2017). Annulus fibrosus was first washed with sterile phosphate-buffered saline (PBS) 3 times and then meticulously eliminated from the human intervertebral disc tissues. The medium was changed every 3 days. Cultured cells were passaged when reaching a confluence of 80–90%. Cells of the second or third generation were used for further assays. Cells were treated with different doses of TNF-α (SRP2102; Sigma-Aldrich, St Louis, MO, United States) for 24 h at 37°C and 95% humidity in an atmosphere of 5% CO_2_. Cells without treatment were used as control.

### Cell Transfection

pLKO.1 lentiviral vectors containing shRNA targeting human METTL14 were synthesized by Sangon Biotech (Shanghai, China). Human METTL14 or SIRT1 ectopic expression vector was constructed using the pLVX-Puro or pcDNA3.1(+) vector, respectively. HEK293T cells were cultured in 6-well plates and those were transfected with pLKO.1-METTL14-shRNA (shRNA), pLVX-Puro-METTL14 (METTL14), or pcDNA3.1(+)-SIRT1 (SIRT1) using the Lipofectamine reagent as per the manufacturer’s protocol. Post-transfection shRNA and METTL14 vectors were collected and were used for transduction. Cells without transduction or transfection were used as control. Cells transduced with pLKO.1-scramble shRNA (shNC), blank pLVX-Puro, or transfected with blank pcDNA3.1(+) vector were used as the negative control.

miR-34a-5p mimic (5′-UGGCAGUGUCUUAGCUGGUUGU-3′), miR-34a-5p inhibitor (5′-ACAACCAGCUAAGACA CUGCCA-3′), and miR-34a-5p negative control (NC, 5′-CAGUACUUUUGUGUAGUACAA-3′) were synthesized by Genepharm Technologies (Shanghai, China). Transfection was performed using Lipofectamine 2,000 (Invitrogen) as per the manufacturer’s instructions. Cells without transfection were used as control.

### Cell Cycle Assay

After treatment, HNPCs were spun down at 1,000 × g for 5 min and fixed with 700 μL of pre-cooled absolute ethanol. RNase A (1 mg/mL, 100 μL) was added to the fixed cells for 30-min incubation 1 in darkness. The resulting cells were further stained with 50 μg/mL of propidium iodide (PI, 400 μL) for 10 min. FACScan flow cytometry (Becton Dickinson, Franklin Lakes, NJ) was then performed using the Cell Quest software (Becton Dickinson).

### SAβ-Galactosidase Staining

After treatment, HNPCs were washed with PBS 3 times and stained with freshly prepared SA-β-Gal staining solution following the manufacturer’s protocol (Beyotime Biotechnology Ltd., Shanghai, China). The stained cells were then observed under a microscope.

### Co-immunoprecipitation

Cell lysates were prepared with the Radioimmunoprecipitation assay (RIPA) lysis buffer. Antibodies, including anti-METTL14 (ab252562; Abcam, Cambridge, MA, United States), anti-DGCR8 (ab90579; Abcam) or normal IgG antibody (sc-2027; Santa Cruz Biotechnology, Inc.), were incubated with the cell lysates followed by incubation with Protein A/G PLUS-Agarose beads (sc-2003; Santa Cruz Biotechnology, Inc.) for 2 h at 4°C. After washing with the lysis buffer 3 times, the samples were subjected to Western blot analysis using anti-METTL14 (ab223090; Abcam) and anti-DGCR8 (ab191875; Abcam) antibodies.

### m6A Content Analysis

m^6^A in total RNA was analyzed with the m6A RNA Methylation Assay Kit as per the manufacturer’s protocol (Abcam, ab185912).

### RNA Immunoprecipitation Assays

Using the Magna RIP RNA-Binding Protein Immunoprecipitation kit (Millipore), RNA immunoprecipitation (RIP) assays were carried out following the manufacturer’s protocol. Total RNA (input control) and isotype control (IgG) were detected simultaneously. RNAs were extracted for reverse transcription and qRT-PCR.

### Luciferase Reporter Assay

SIRT1 3′-UTR region containing a putative miR-34a-5p binding site was cloned into the pGL3 vector. For SIRT1 luciferase reporter assay, the HNPCs were transfected with miR-34a-5p mimic, miR-34a-5p inhibitor, and pGL3-SIRT1-WT or pGL3-SIRT1-MUT plasmid and pRL-TK vector (Promega) expressing the renilla luciferase with Lipofectamine 2,000. The relative luciferase activity was determined and normalized to the Renilla luciferase activity 48 h after transfection according to the standard protocol.

### RNA Isolation and Quantitative RT-PCR

Total RNA of HNPCs was extracted using TRIzol reagent (Invitrogen) and the RNeasy Mini Kit (Qiagen, Hilden, Germany). Reverse transcription was conducted using PrimeScript^TM^RT reagent Kit with gDNA Eraser (Takara, Beijing, China). The resulting products were used for qRT-PCR amplification with SYBR Premix Ex Taq^TM^ GC (Takara). GAPDH or U6 was used as the normalization control. The primers for qRT-PCR are listed in Table 1. The fold changes of mRNA or miRNA were determined by the 2^–ΔΔ*CT*^ method.

**TABLE 1:** Primes sequences used in this study.

**Gene**	**Sequences (5′–3′)**
TNF-α-forward	GGTATGAGCCCATCTATCTG
TNF-α-reverse	AGGGCAATGATCCCAAAG
METTL3-forward	CCTTTGCCAGTTCGTTAGTC
METTL3-reverse	TCCTCCTTGGTTCCATAGTC
METTL14-forward	CTGGGAATGAAGTCAGGATAG
METTL14-reverse	CCAGGGTATGGAACGTAATAG
WTAP-forward	AAAGCAGTGAGTGGGAAAG
WTAP-reverse	AGCGGCAGAAGTATTGAAG
SIRT1-forward	ACCTCCTCATTGTTATTGG
SIRT1-reverse	TTACAGGGTTACAGCAAAG
pri-miR-34a-forward	AGTTGCTGAAGGTGGTGGTC
pri-miR-34a-reverse	ACATGCGTGCCTGTAGTCC
GAPDH-forward	AATCCCATCACCATCTTC
GAPDH-reverse	AGGCTGTTGTCATACTTC
miR-200c-3p-forward	CGCGTAATACTGCCGGGTAAT
miR-200c-3p-reverse	AGTGCAGGGTCCGAGGTATT
miR-27a-3p-forward	GCGCGTTCACAGTGGCTAAG
miR-27a-3p-reverse	AGTGCAGGGTCCGAGGTATT
miR-34a-5p-forward	CGCGTGGCAGTGTCTTAGCT
miR-34a-5p-reverse	AGTGCAGGGTCCGAGGTATT
miR-15b-5p-forward	CGCGTAGCAGCACATCATGG
miR-15b-5p-reverse	AGTGCAGGGTCCGAGGTATT
pre-miR-34a-forward	CCTAGAAGTGCTGCACGTTGTG
pre-miR-34a-forward	AGTGCAGGGTCCGAGGTATT
U6-forward	CTCGCTTCGGCAGCACA
U6-reverse	AACGCTTCACGAATTTGCGT

### Western Blot

The cell lysates were prepared using the radioimmunoprecipitation assay lysis buffer (Beyotime, Shanghai, China). The quantitation of protein in the samples was performed using the bicinchoninic acid (BCA) Protein Assay Kit (Beyotime). For SDS-PAGE, 20 μg of protein sample was loaded to each well followed by the transmembrane onto polyvinylidene fluoride membranes (Millipore, Billerica, MA, United States). The blocking of the membrane was conducted by incubating with 5% fat-free milk and then primary antibodies against METTL3 (ab195352; Abcam), METTL14 (ab223090; Abcam), WTAP (ab195380; Abcam), SIRT1 (ab110304; Abcam), and GAPDH (#5174, Cell Signaling Technology) overnight at 4°C. The blots were then washed in Tris-buffered Saline Tween-20 (TBST) three times and then incubated with secondary antibodies (A0208, A0216; Beyotime, Shanghai, China). The blots were examined by chemiluminescence using the Enhanced Chemiluminescence Detection kit (Pierce Biotechnology, Rockford, IL, United States). After exposure, the intensity of bands was analyzed by Image-Pro Plus 6.0 software.

### Statistical Analysis

All statistical analyses were performed using GraphPad Prism 8.0.2 (GraphPad Software, San Diego, CA, United States). Data were shown as mean ± standard deviation (SD) from the triplicates of independent experiments. The difference between different groups was analyzed using a two-sided Student’s *t*-test and ANOVA. *P-*values< 0.05 were considered significant.

## Results

### The Level of m6A Modification Is Positively Correlated With TNF-α in IVDD Patients

**FIGURE 1:**
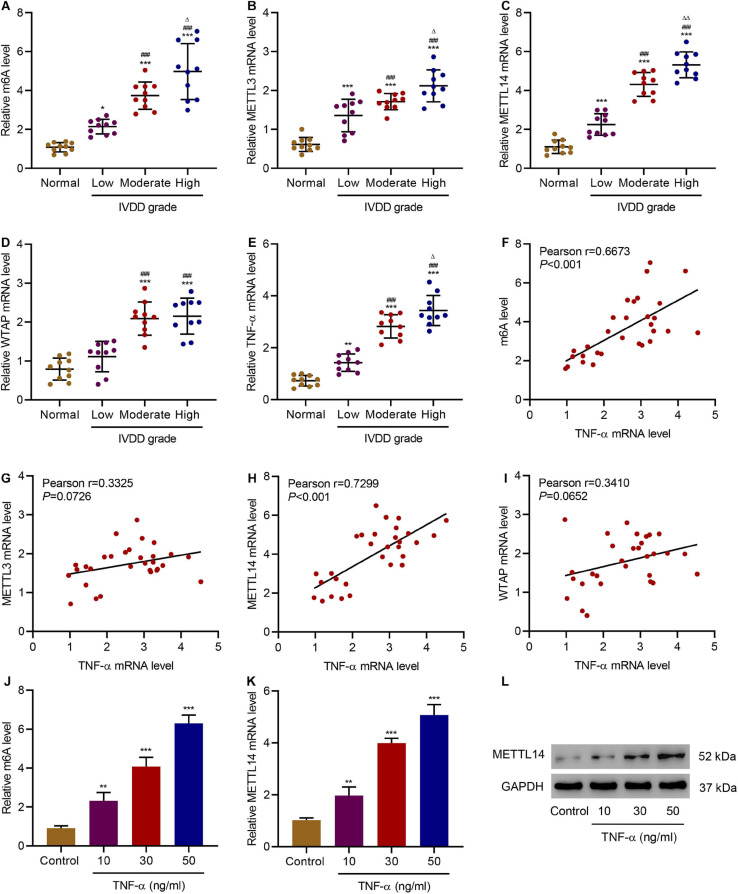
Correlation between TNF-α and m6A modification in IVDD patients. **(A)** The m6A level and expression of **(B)** METTL3, **(C)** METTL14, **(D)** WTAP, and **(E)** TNF-α in normal controls and IVDD patients. **(F–I)** Pearson correlation scatter plots in IVDD patients (*n* = 30). **(J)** The m6A level and **(K,L)** expression of METTL14 in HNPCs treated with different doses of TNF-α. *P < 0.05, **P < 0.01, ***P < 0.001 compared with normal control; ^###^*P* < 0.001 compared with low grade; ^Δ^ *P* < 0.05, ^ΔΔ^ *P* < 0.01 compared with moderate grade.

TNF-α is the main pro-inflammatory cytokines principally associated with the progression of IVDD (Purmessur et al., 2013). To verify the relationship between TNF-α and the m6A methylation in IVDD patients, we first examined the level of m6A methylation in the NP tissues from the patients and discovered an increase in the level of m6A modification (Figure 1A). Therefore, we further implemented screening of the expression of genes related to m6A methylation, including *METTL3*, *METTL14*, and *Wilms tumor 1-associated protein* (Figures 1B–D). With the increase of the IVDD grades, all the genes exhibited higher expression than the normal group. Meanwhile, TNF-α expression increased along with the IVDD grades (Figure 1E). To better determine the correlation between the expression of TNF-α and m6A modification-related genes, we calculated Pearson correlation coefficients for each gene by plotting the TNF-α mRNA level against the m6A level or the mRNA level of the genes (Figures 1F–I). Among the three genes, *TNF-*α and *METTL14* (*r* = 0.7299, *P* < 0.001) were more correlated than the other two, which is similar to the correlation between TNF-α expression and the m6A methylation level. Similar phenomena were also observed in HNPCs. With the increase in the stimulation concentrations of TNF-α, the level of m6A methylation was significantly elevated (Figure 1J). Accordingly, compared with the untreated HNPCs, the METTL14 mRNA level and the protein level in the treatment groups had also experienced significant increases (Figures 1K,L). As a result, it is indicated that the METTL14-mediated m6A modification is positively correlated with TNF-α in IVDD models.

### METTL14 Knockdown Inhibits TNF-α-Induced Cell Cycle Arrest and Senescence

**FIGURE 2:**
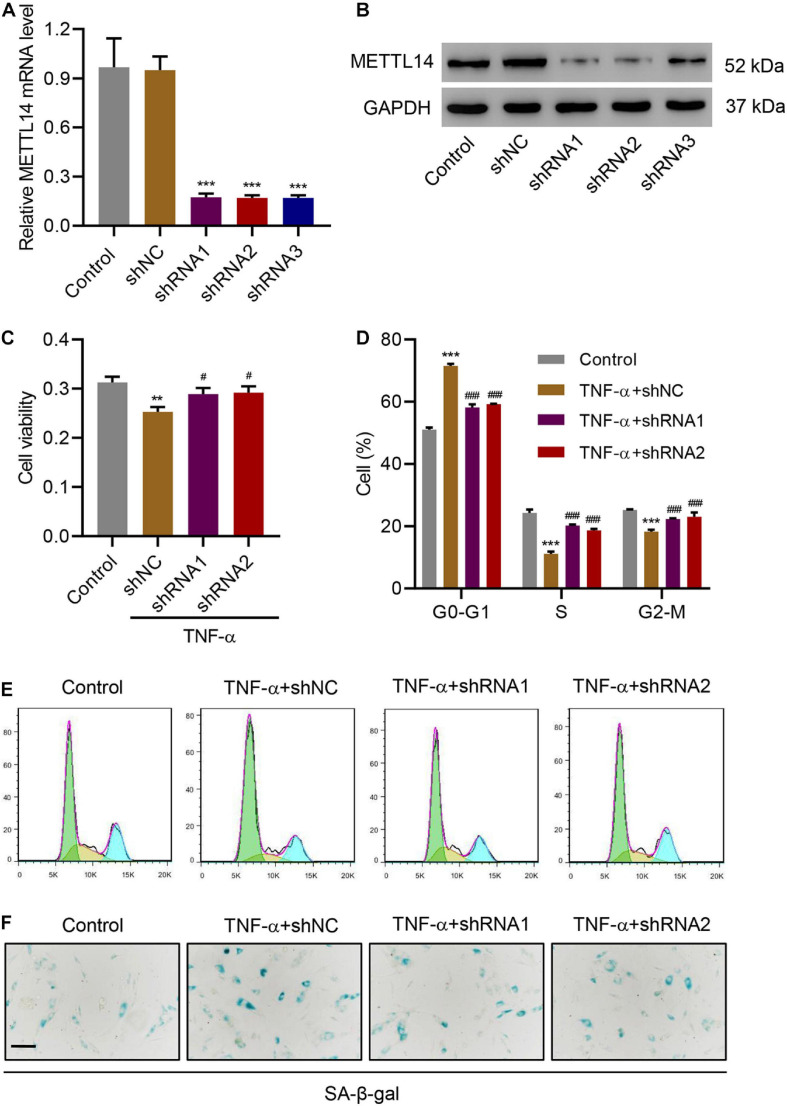
METTL14 silencing inhibits TNF-α-induced cell cycle arrest and senescence. **(A,B)** Expression of METTL14 in HNPCs transduced with METTL14 shRNA vectors. **(C)** Cell viability, **(D,E)** cell cycle, and **(F)** SA-β-gal staining of HNPCs transduced with METTL14 shRNA vectors and treated with 30 ng/mL TNF-α for 24 h. Scale bar: 50 μm. **P < 0.01, ***P < 0.001 compared with control; ^###^*P* < 0.001 compared with TNF-α + shNC.

The discovery of the positive correlation between the expression of METTL14 and TNF-α in IVDD models motivated us to further explore the impact of METTL14 on TNF-α-induced NP cellular processes. We developed three shRNAs (shRNA 1-3) to silence METTL14 in HNPCs, which significantly reduced the expression of the mRNA and protein compared with the controls (Figures 2A,B). Especially, shRNA 1 and 2 showed better inhibitory potency according to the Western blot result (Figure 2B). In the cell viability assay, TNF-α treatment reduced the cell viability while the use of the two shRNAs to TNF-α-treated HNPCs significantly restored the cell viability (Figure 2C). A further investigation indicated that the suppression of the METTL14 expression affected the TNF-α-induced cell cycle arrest (Figures 2D,E). TNF-α caused the cell cycle arrest of HNPCs at the G0-G1 stage. In comparison, shRNA1 and 2 significantly drove the cells to enter the S and G2-M phases. Additionally, the knockdown of METTL14 enabled the inhibition of TNF-α-induced cell senescence (Figure 2F). Using a senescence-associated (SA) β-galactose assay, we observed that the blue-dyed precipitates had been considerably diminished in the shRNA-treatment groups. From the results above, it is suggested that METTL14 knockdown significantly inhibits TNF-α-induced cell viability inhibition, cell cycle arrest, and cell senescence.

### METTL14 Overexpression Promotes Cell Cycle Arrest and Senescence

**FIGURE 3:**
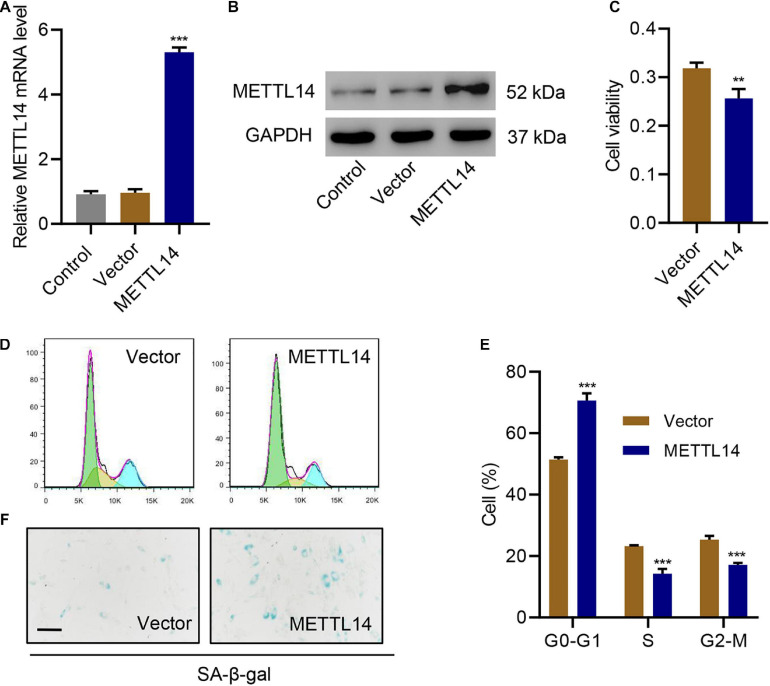
METTL14 overexpression promotes cell cycle arrest and senescence. **(A,B)** Expression of METTL14 in HNPC cells transduced with the METTL14 expression vector. **(C)** Cell viability, **(D,E)** cell cycle, and **(F)** SA-β-gal staining of HNPCs transduced with a METTL14 expression vector for 24 h. Scale bar: 50 μm. **P < 0.01, ***P < 0.001 compared with vector.

To further evaluate the influence of METTL14 on cell cycle arrest and senescence, we overexpressed METTL14 in HNPCs without the TNF-α treatment (Figures 3A,B). Interestingly, despite the absence of TNF-α, the overexpression of METTL14 led to reduced cell viability (Figure 3C). Likewise, METTL14 overexpression remarkably influenced the cell cycle (Figures 3D,E). METTL14 overexpression enhanced the cell cycle arrest, which brought up the percentage of the cells of the G0-G1 phase by 20% (50% for Vector vs. 70% for METTL14). Accordingly, the cell percentage of S and G2-M phases declined. With regard to the efficiency, the overexpression of METTL14 was even comparable to the TNF-α treatment (Figures 3D,E). When using the SA β-galactose assay to identify the cell senescence of METTL14-overexpressing HNPCs, we observed promoted cell senescence (Figure 3F), which was similar to the TNF-α treatment. Therefore, the METTL14 overexpression assays substantiated that METTL14 inhibited cell viability and promote cell cycle arrest and senescence.

### METTL14-Dependent m6A Methylation Regulates the Processing of miR-34a by DGCR8

**FIGURE 4:**
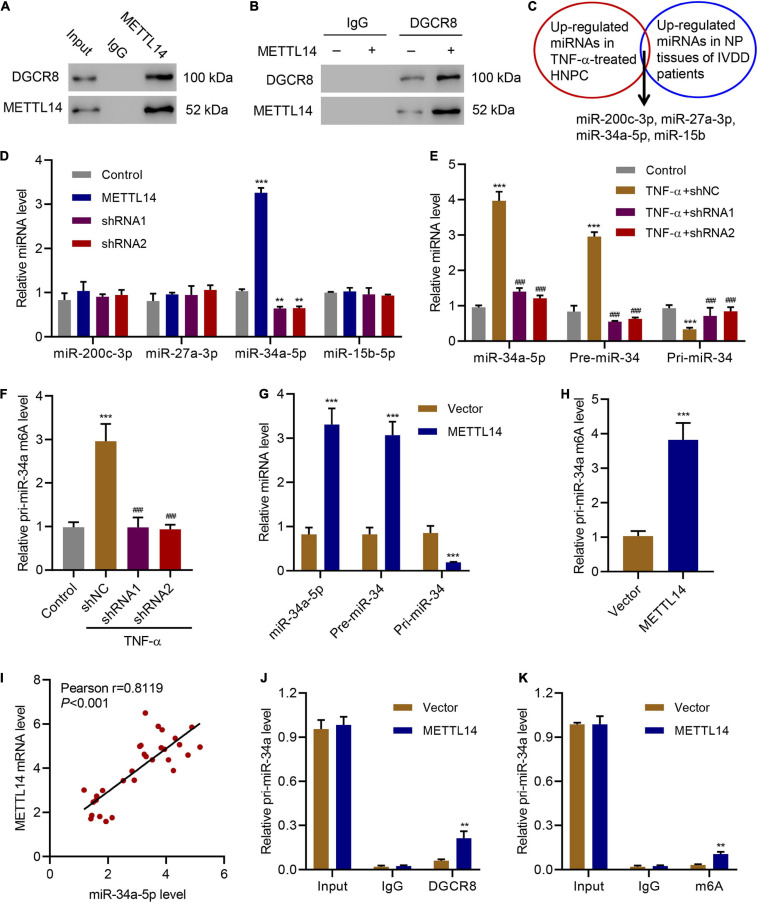
METTL14-dependent m6A methylation regulates the processing of miR-34a by DGCR8. **(A)** Co-immunoprecipitation of the METTL14-interacting protein DGCR8. IgG antibody was used as the control for the immunoprecipitation. **(B)** Immunoprecipitation of DGCR8 in cells overexpressing METTL14 or not. Western blot was conducted using the antibodies depicted. **(C)** Four up-regulated miRNAs were searched out according to their expression levels in TNF-α-treated HNPC cells and in NP tissues of IVDD patients. **(D)** Expression of miR-200c-3p, miR-27a-3p, miR-34a-5p and miR-15b-5p in HNPC cells transduced with METTL14 shRNA vectors or overexpression vector. **(E)** Expression of pri-miR-34a, pre-miR-34a, and miR-34a-5p and **(F)** the levels of pri-miR-34a m6A in HNPC cells transduced with METTL14 shRNA vectors and treated with 30 ng/mL TNF-α for 24 h. **(G)** Expression of pri-miR-34a, pre-miR-34a, and miR-34a-5p and **(H)** the levels of pri-miR-34a m6A in HNPC cells transduced with a METTL14 expression vector for 24 h. **(I)** Pearson correlation scatter plots in IVDD patients (*n* = 30). Immunoprecipitation of **(J)** DGCR8-associated and **(K)** m6A modified RNA from HNPC cells transduced with METTL14 expression vector followed by qRT-PCR to detect pri-miR-34 binding to DGCR8 and to assess the pri-miR-34a m6A modification level, respectively. **P < 0.01, ***P < 0.001 compared with control or vector. ^###^*P* < 0.001 compared with TNF-α + shNC.

METTL14 can interact with DGCR8 to positively regulate the primary miRNA process in an m6A-dependent manner (Ma et al., 2017). In our case, we intended to pursue the downstream target of the METTL14-DGCR8 axis. To this end, we first established the interaction between DGCR8 and METTL14 in HNPCs by co-immunoprecipitation (Figure 4A). Moreover, in METTL14-overexpressing HNPCs we also saw a significant increase in the binding between METTL14 and DGCR8 (Figure 4B). The findings above solidified that METTL14 mediated pri-miRNA processing by regulating the recognition and binding of DGCR8 to pri-miRNAs.

Furthermore, we detected the association between the four miRNAs, which were upregulated in TNF-α-treated HNPCs and in NP tissues from IVDD patients (Cao and Chen, 2017; Kang et al., 2017; Cheng et al., 2018; Xiang et al., 2020), and METTL14 (Figure 4C). Compared with the other three miRNAs, the miR-34a-5p level skyrocketed (>3.5-fold) accompanying the overexpression of METTL14 (Figure 4D). Accordingly, the suppression of METTL14 by the specific shRNAs significantly reduced the miR-34a-5p relative level. When subjected to the TNF-α treatment, HNPCs exhibited remarkably high expression of miR-34a-5p and pre-miR-34 but low level of pri-miR-34, indicating that miR-34 was processed (Figure 4E). The addition of METTL14 shRNAs tremendously mitigated the TNF-α-induced increase in mRNA levels of miR-34a-5p and pre-miR-34. Furthermore, the relative pri-miR-34a m6A level was also enhanced by TNF-α, which was abolished by METTL14 knockdown (Figure 4F). Conversely, the overexpression of METTL14 promoted the formation of miR-34a-5p and pre-miR-34 (Figure 4G). The relative level of pri-miR-34a m6A showed a fourfold increase when METTL14 was overexpressed (Figure 4H).

As a result, the level of METTL14 exhibited a positive correlation with that of miR-34a-5p in NP tissues of IVDD patients (Figure 4I). The regulation of the pri-miR-34a level was tightly associated with the interaction between METTL14 and DGCR8 (Figures 4J,K). The DGCR binding level as well as the m6A modification level was significantly promoted by METTL14, indicating that METTL14 played a vital role in the maturation of pri-miR-34a. Taken together, these results indicate that the METTL14 promoted the processing of pri-miR-34a by DGCR8 in an m6A manner.

### miR-34a-5p Inhibitor Rescues the Cell Cycle Arrest and Senescence Induced by METTL14 Overexpression

**FIGURE 5:**
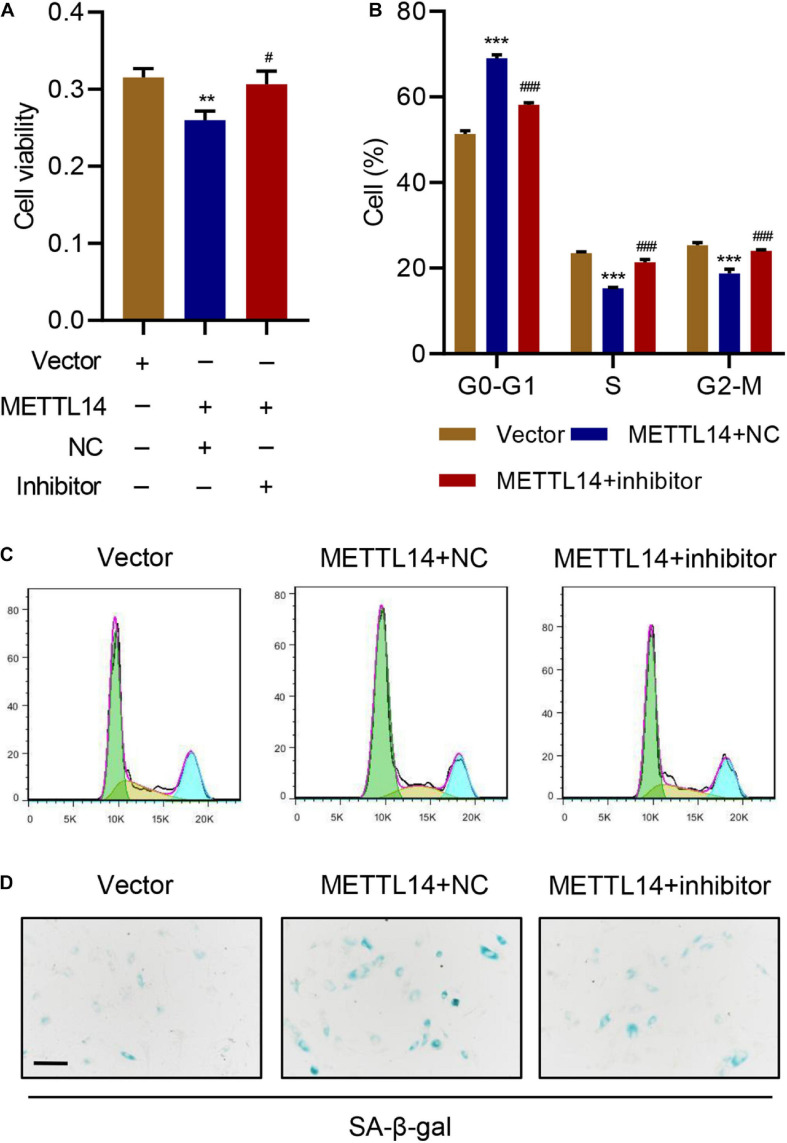
miR-34a-5p inhibitor rescues the cell cycle arrest and senescence induced by METTL14 overexpression. **(A)** Cell viability, **(B,C)** cell cycle, and **(D)** SA-β-gal staining of HNPC cells transduced with the METTL14 expression vector and transfected with miR-34a-5p inhibitor for 24 h. Scale bar: 50 μm. **P < 0.01, ***P < 0.001 compared with vector. ^###^*P* < 0.001 compared with METTL14 + NC.

To verify the potential function of miR-34a-5p in IVDD, we introduced the corresponding miRNA inhibitor in the METTL14-overexpressing HNPCs. The miR-34a-5p inhibitor considerably counteracted the effects of METTL14 overexpression on cell senescence of HNPCs (Figure 5). The cell viability (Figure 5A), cell cycle arrest (Figures 5B,C), and cell senescence (Figure 5D) were largely rescued by the inhibitor. As a result, evidenced by the data, the METTL14-induced cell cycle arrest and senescence can be recovered by the miR-34a-5p inhibitor, thus manifesting the indispensable role of miR-34a-5p in regulating METTL14-dependent cell senescence.

### miR-34a-5p Promotes Cell Cycle Arrest and Senescence by Targeting SIRT1

**FIGURE 6:**
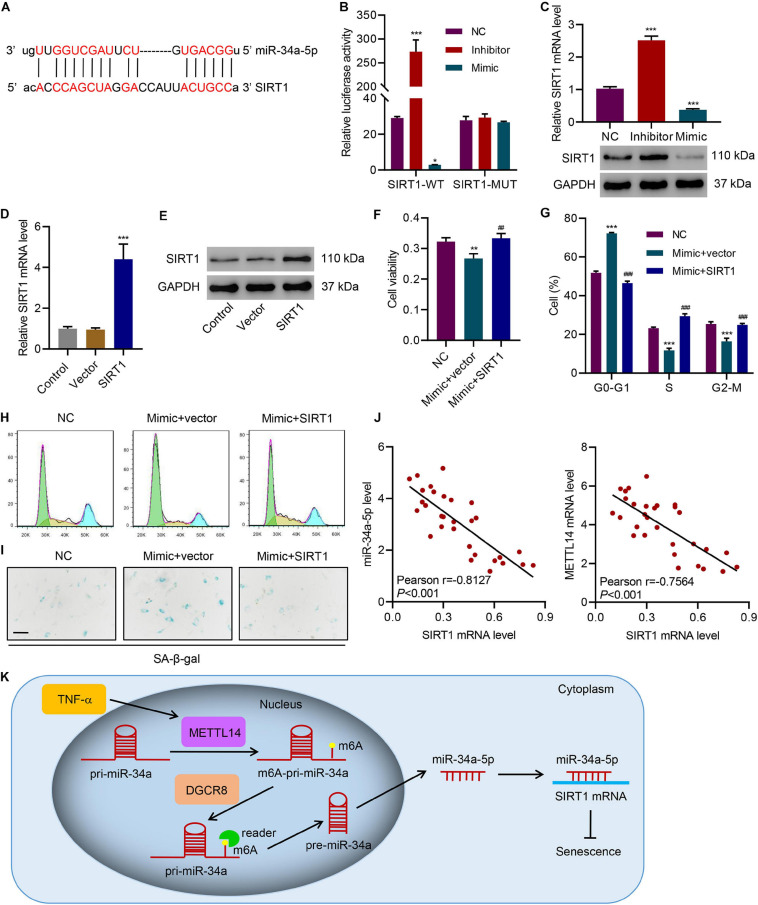
miR-34a-5p promotes cell cycle arrest and senescence by targeting SIRT1. **(A)** Predictive miR-34a-5p binding sites in the 3′-UTR of SIRT1 mRNA. **(B)** Dual-luciferase reporter assays demonstrated that SIRT1 was the direct target of miR-34a-5p. **(C–E)** Expression of SIRT1 in HNPC cells transfected with miR-34a-5p inhibitor, miR-34a-5p mimic, or SIRT1 expression vector. **(F)** Cell viability, **(G,H)** cell cycle, and **(I)** SA-β-gal staining of HNPC cells transfected with miR-34a-5p mimic and a SIRT1 expression vector for 24 h. **(J)** Pearson correlation scatter plots in IVDD patients (*n* = 30). Scale bar: 50 μm. **(K)** Diagram of the mechanism. *P < 0.05, **P < 0.01, ***P < 0.001 compared with NC or vector. ^##^*P* < 0.01, ^###^*P* < 0.001 compared with mimic + vector.

Using the mRNA interaction prediction server^1^, we predicted the potential interaction between the miR-34-5p and the 3′-UTR of SIRT1 mRNA (Figure 6A). To better analyze the interaction, we devised a dual-luciferase assay using the SIRT1 wildtype mRNA (SIRT1-WT) and the SIRT1 3′-UTR mutant mRNA (SIRT1-MUT) in HNPCs that were treated with the miR-34a-5p inhibitor or the miR-34a-5p mRNA mimic (Figure 6B). In the SIRT1-WT group, the inhibitor approximately generated a 9-fold drastic increase in luciferase activity. By contrast, the mimic led to lower luciferase activity. Instead of inducing changes in luciferase activities, in the SIRT1-MUT group, no matter whether the inhibitor or the mimic failed to generate any signal, indicating that the mutation of 3′-UTR jeopardized the interaction between miR34a-5p and SIRT1 mRNA. Furthermore, SIRT1 mRNA expression can be influenced by miR-34a-5p (Figure 6C). The miR-34a-5p inhibitor significantly promoted SIRT1 expression in HNPCs while the mimic considerably suppressed the expression. We next overexpressed SIRT1 in HNPCs to determine the effects of SIRT1 on cell senescence of HNPCs in the presence of the inhibitor or the mimic of miR-34a-5p (Figures 6D,E). The miR-34a-5p mimic lowered the cell viability of HNPCs. However, SIRT1 overexpression in HNPCs largely restored the cell viability despite the presence of the miR-34a-5p mimic (Figure 6F). SIRT1 overexpression was also active in attenuating the miR-34a-5p-induced cell cycle arrest, in which more HNPCs entered S and G2-M phases (Figure 6G,H). Cell senescence of HNPCs caused by miR-34a-5p was reversed by SIRT1 overexpression as well (Figure 6I). Based on these findings, we established the relationship between the level of SIRT1 mRNA and the levels of miR-34a-5p and METTL14 mRNA in NP tissues of IVDD patients (Figures 6J,K). SIRT1 expression was negatively correlated with either the miR-34a-5p level or the METTL14 mRNA level. Therefore, our results demonstrate that SIRT1 served as a critical target in process of the miR-34a-5p-promoted cell cycle arrest and senescence.

## Discussion

miRNA has been found closely associated with cellular process regulation, cell function, and diseases (Bhaskaran and Mohan, 2014). The modification of miRNAs, especially methylation, can largely affect the functions of mRNA, which further regulates cellular processes and biological activities (Bianchi et al., 2017). Recent reports link IVDD with miRNAs, suggesting that miRNAs can act as potential therapeutic targets (Li et al., 2015; Zhou et al., 2017). Herein, we have devised various assays to clarify the mechanism of m6A methylation promoted cell senescence in IVDD.

The level of m6A modification significantly increased in the HNPCs, which was correlated with the level of TNF-α in IVDD patients. m6A modification has been characterized as the most prevalent internal mRNA modification in mammalian cells, which accounts for regulating various important biological processes (Zhang et al., 2019). Rising evidence is confirming the role of m6A in cell development and cancers (Fazi and Fatica, 2019; Chen and Wong, 2020). Li et al. have characterized the function of m6A methylation in controlling the proliferation of human glioma cells by influencing apoptosis (Li F. et al., 2019). Yang et al. have reported the m6A-modulated proliferation and apoptosis of lens epithelial cells (Yang et al., 2020). However, there is no previous report on the relation between m6A methylation and IVDD. Our discovery of a high level of m6A modification in NP tissues of IVDD patients expands the scope of related research. More importantly, we have identified that METTL14, one of the “writer” protein, was more tightly associated with m6A modification, the expression of which was positively correlated with the level of m6A methylation as well as the TNF-α. Jian et al. (2020) have reported the mechanism of METTL14-promoted endothelial inflammation and atherosclerosis through driving FOXO1 m6A modifications. They proved the major role of METTL14 in TNF-α-induced endothelial cell inflammation. As a process tightly associated with inflammation, cell senescence includes irreversible cell cycle arrest (Stojanovic et al., 2020). In our study, we have clearly illustrated the role of METTL14 in cell senescence, which solidifies the function of METTL14 in TNF-α-induced inflammation. With the increased expression of this writer protein, cell viability decreased while cell cycle arrest and senescence were significantly promoted.

The identification of METTL14 as the main regulator of m6A modification in IVDD models allowed us to further explore the mechanism behind it. Studies have shown that METTL14 actively participates in the processing of miRNAs by interacting with DGCR8 (Feng et al., 2010; Ma et al., 2017). Accordingly, our co-immunoprecipitation assay also confirmed the interaction between METTL14 and DGCR8 in HNPCs, which proves that METTL14 played a role in regulating miRNA maturation in IVDD models. Interestingly, through an in-depth screening of miRNA change in either HNPCs or NP tissues of IVDD patients, we identified several upregulated miRNAs, in which miR-34a-5p showed a positive correlation with the m6A modification level. When varying the METLL14 level in HNPCs, we were able to capture the regulation of the miR-34a-5p processing. Similar to the processing of miR-126 (Feng et al., 2010) and miR-19a (Zhang et al., 2020), METLL14 positively modulated the maturation of miR-34. Thus, the RNA levels of pre-miR-34 and miR-34a-5p were significantly elevated in contrast to the mitigated pri-miR-34 level. The m6A methylation of pri-miR-34 was found active under the circumstance of METTL14 overexpression.

Previous studies have discussed the mechanism of m6A-promoted cell senescence (Li Q. et al., 2017; Wu et al., 2020). The METTL3/METTL14-mediated m6A methylation can enhance p21 expression which is further promoted. oxidative stress-induced cellular senescence (Li Q. et al., 2017). Through interacting with Lamin A, METTL3/14 can be properly localized in the nuclear speckles to achieve the regulatory function (Wu et al., 2020). Our study has deepened the current understanding of m6A modification in cell senescence. Mainly regulated by METTL14, m6A-involved cell senescence was originated from the methylation of miR-34a-5p followed by the interaction with the 3′-UTR of the SIRT mRNA. During the process, the maturation of the mRNA was largely promoted, reflected by the escalated levels of miR-34a-5p and pre-miR-34and reduced level of pri-miR-34. The role of METTL3 in the regulation of senescence.

It is noted that miRNAs can play vital roles in cell senescence (Li et al., 2009; Faraonio et al., 2012; Baker et al., 2019). miR-34a-5p has been identified as a possible cause of cell senescence in HNPCs in our study. Xia et al. have characterized the mechanism of miR-34a-5p-induced cardiac senescence-related injury. It is shown that miR-34a-5p serves as an exosomal transfer RNA and the inhibition of miR-34a-5p mitigated the pro-senescent effect in cardiomyocytes and subsequently alleviated the irreversible cell cycle arrest (Xia et al., 2020). miR-34a-5p is also found involved in regulating the switch between senescence and apoptosis in non-small cell lung cancer (Gupta et al., 2020). Herein, we have presented that the inhibition of miR-34a-5p significantly decreases the senescence of HNPCs. As a result, miR-34a-5p demonstrates prevalent senescent effects in varieties of tissues and organs, which can be a major biological function of this miRNA.

Additionally, Maes et al. (2009) used a miRNA microarray assay to reveal the upregulation of miR-34 in senescent cells. We advanced the results and proved that this miRNA was further processed through the METTL14-DGCR8 axis. Our data have demonstrated that miR-34a-5p induced cell senescence by targeting the downstream factor SIRT1. SIRT1 localizes in both the nucleus and cytoplasm to function in many crucial biological activities, including lifespan extension, ADP-ribosyl-transferase, DNA repair, cell cycle arrest, and cellular senescence (Lee et al., 2019). The sequence of miR-34a-5p was predicted to pair with that of the 3′-UTR of SIRT1. The use of the miR-34a-5p mimic resulted in typical senescence that was largely inhibited by overexpressing SIRT1. In IVDD patients, SIRT1 expression was negatively correlated with the levels of the METTL14 mRNA and miR-34a-5p. Therefore, our findings reinforce the idea that SIRT1 prevents cell senescence.

Our data were mainly obtained from the *in vitro* model of HNPCs, which can be further explored in *in vivo* IVDD models. Due to limited studies in the related field, the concrete mechanism of METTL14-mediated m6A modification and maturation of miR-34a has not been fully elucidated. Efficient disruption of the interaction between miR-34a-5p and SIRT1 in both *in vitro* and *in vivo* levels to alleviate IVDD still requires more sophisticated and intensive investigations. Additionally, the interaction between METTL14 and DGRC8 is also worth dedicated studies to uncover the entire axis.

Our current study reveals that m6A-modified miR-34a-5p promotes induced NPC senescence by targeting SIRT1, which represents the first attempt to discover the association between miRNA modification and cell senescence in IVDD models (Figure 6K). By characterizing the role of the miR-34a-5p-SIRT1 axis in cell senescence, we have proposed a potential direction for developing an IVDD therapy by disrupting the interaction between miR-34a-5p and SIRT1 or inhibiting METTL14.

## Data Availability Statement

The original contributions presented in the study are included in the article/supplementary material, further inquiries can be directed to the corresponding author/s.

## Ethics Statement

The studies involving human participants were reviewed and approved by The Affiliated Shanghai General Hospital of Nanjing Medical University. The patients/participants provided their written informed consent to participate in this study.

## Author Contributions

HZ and BS conceived and designed the work. BS, LZ, and GZ performed the research and collected and analyzed the data. HZ and QS collected human tissue samples and wrote the manuscript. BS, LZ, and QS provided technical assistance. All authors read and approved the final manuscript.

## Conflict of Interest

The authors declare that the research was conducted in the absence of any commercial or financial relationships that could be construed as a potential conflict of interest.

## Footnotes

1 http://www.microrna.org/
